# Medial Thigh Cutaneous Flap Irrigated by Perforating Vessels of the Secondary Pedicle of the Gracilis Muscle: Anatomical Study and Clinical Case Report

**DOI:** 10.1055/s-0045-1809398

**Published:** 2025-07-15

**Authors:** Gabriel Vique Valeriano, Antonio Carlos da Costa, Diego Figueira Falcochio, Yussef Ali Abdouni

**Affiliations:** 1 Hand Surgery and Microsurgery Group, Department of Orthopedics and Traumatology, Irmandade da Santa Casa de Misericórdia de São Paulo (DOT/ISCMSP), São Paulo, SP, Brazil

**Keywords:** gracilis muscle, surgical flaps, thigh, coxa, músculo grácil, retalhos cirúrgicos

## Abstract

**Objective:**

To analyze the anatomy of the perforating vessels irrigating the skin of the medial aspect of the thigh and originating from the secondary vascular pedicle of the gracilis muscle.

**Methods:**

We dissected 33 thighs from cadavers of both sexes to record and analyze the characteristics of the blood vessels irrigating the gracilis muscle in the middle third of the thigh.

**Results:**

All thighs (100%) had a secondary vascular pedicle in the intermuscular septum between the gracilis and vastus medialis/sartorius muscles, with branches irrigating the overlying skin on the medial aspect. The pedicle presented an arterial diameter ranging from 1.3 to 4.6 mm (mean: 2.3 mm) and a length ranging from 28 to 84 mm (mean: 50.6 mm). In 87.8% of the cases, the pedicle irrigated the gracilis muscle and presented vessels to the overlying skin.

**Conclusion:**

Based on the data found, we conclude that it is feasible to obtain a reliable flap from the medial aspect of the middle third of the thigh.

## Resumo

**Objetivo:**

Estudar a anatomia dos vasos perfurantes que irrigam a pele da face medial da coxa, provenientes do pedículo vascular secundário do músculo grácil.

**Métodos:**

Foram dissecadas 33 coxas de cadáveres de ambos os sexos, e registradas e analisadas as características dos vasos sanguíneos que irrigam o músculo grácil no terço médio.

**Resultados:**

Em todas as coxas (100%) foi evidenciada a presença de um pedículo vascular secundário no septo intermuscular entre os músculos grácil e vasto medial/sartório, com ramos que irrigam a pele suprajacente na face medial. O pedículo apresentou um diâmetro arterial que variou de 1,3 a 4,6 mm (média de 2,3 mm) e comprimento entre 28 e 84 mm (média de 50,6 mm). Em 87,8% dos casos, o pedículo irrigava o músculo grácil e apresentava vasos para a pele suprajacente.

**Conclusão:**

Com base nos dados encontrados, podemos concluir que é possível obter um retalho confiável da face medial do terço médio da coxa.

## Introduction

Injuries resulting from high-energy trauma, mainly traffic accidents, often involve soft tissue loss and exposure of essential structures, such as bones, tendons, vessels, and nerves. 1 These therapeutic situations are difficult to manage and challenging for the reconstructive surgeon.

The improvements in surgical techniques have increased treatment success rates. Today, free flaps are an effective option among reconstructive techniques. Perforator flaps are an evolution of microsurgical flaps, enabling the creation of thinner flaps and resulting in better functional and esthetic outcomes. 2

In 1967, Fujino 3 documented the distribution of perforating arteries to supply the interstitium, similar to axial arteries. In 1988, Koshima et al. 4 pioneered perforating artery-based flaps exclusively consisting of skin and subcutaneous tissue. Wei et al. 5 defined perforating artery-based flaps as those supplied by arteries crossing the adjacent deep fascia and making it feasible to dissection through the muscle or the intermuscular septum to their vessel of origin, with no need for muscle inclusion in the flap.

Focusing on the medial aspect of the thigh, certain authors, such as Peek et al. 6 and Eom et al., 7 described, in anatomical studies and case reports, a flap from this region based on the perforating arteries originating from the main vascular pedicle of the gracilis muscle. This flap has been widely studied and may be used in its pedicled or free form to cover different defects in the inguinal region or distant areas.

Feng et al., 8 Scaglioni et al., 1 and Zheng et al. 9 also described, through anatomical studies and some clinical cases, the anteromedial thigh flap or lower medial thigh flap based on perforating branches from muscular arteries irrigating the vastus medialis muscle. In 1984, Song et al. 10 described the perforating flap from the deep femoral artery, harvested from the posteromedial aspect of the thigh.

The gracilis muscle, in the medial region of the thigh, is commonly used as a functional free muscle flap. Mathes and Nahai 11 classified its irrigation pattern as type 2, that is, muscle irrigation occurs by a dominant vascular pedicle, able to supply the entire muscle, but with the contribution of several smaller secondary pedicles. Some branches of these vessels cross the deep fascia in the medial region of the thigh and irrigate the subcutaneous tissue and overlying skin. In an anatomical study with arteriography, Peek et al. 6 identified the presence of a secondary pedicle in the intermediate region of the thigh with multiple intramuscular anastomoses with the main vascular pedicle.

The objective of the present study was to evaluate the anatomical parameters of the vessels irrigating the skin and fascia of the medial region of the thigh, originating from the secondary vascular pedicle of the gracilis muscle in the middle third of the thigh, defining its location, diameter, distribution pattern, and anatomical variations, showing the feasibility of obtaining a reliable flap based on this vascular pedicle.

## Materials and Methods

The institutional Ethics Committee approved the study under number CAAE: 15621319.1.0000.5479.

We performed microsurgical dissection of 33 thighs from 18 cadavers. In total, 19 thighs were from male cadavers, and 14, from female cadavers. We recorded biometric data of the cadavers, such as age, height, and weight. We excluded cadavers with peripheral vascular disease as the known cause of death and those with scars in the dissection region.

The same surgeon performed all dissections using a surgical magnifying glass with 3.5x magnification. We recorded anatomical parameters of the vascular pedicle, such as vessel length, diameter, location, origin, and distribution, in addition to their relationship with thigh length. We obtained the measurements using a millimeter tape and a digital four-dimensional 150-mm caliper (Digimess Instrumentos de Precisão Ltda.).

The Kolmogorov-Smirnov test assessed the normal distribution of the quantitative outcome variables. The analysis of quantitative variables used the equality of two proportions and Pearson's correlation tests. The statistical confidence interval was of 95%.

## Dissection

**Fig. 1:**
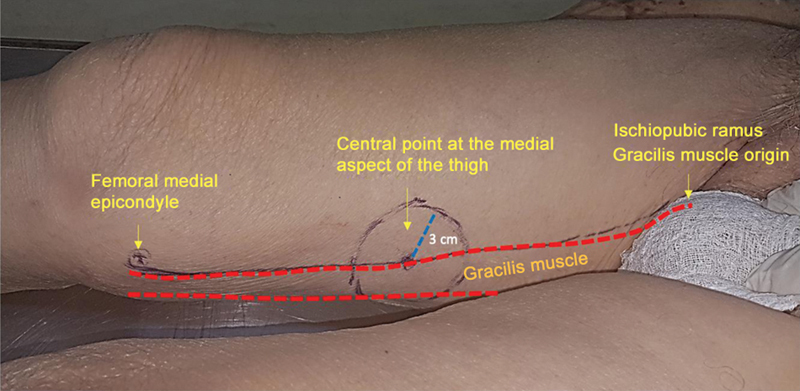
Medial aspect of the right thigh with a drawing showing the location of the vascular pedicle.

**Fig. 2:**
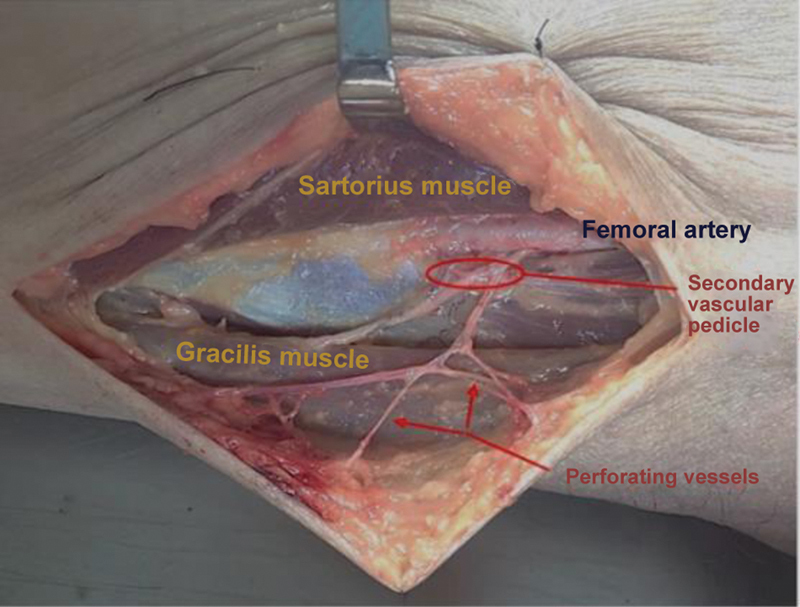
Dissection of the secondary vascular pedicle of the gracilis muscle with its perforating vessels.

Initially, we drew a line on the medial aspect of the thigh, from the ischiopubic ramus to the medial femoral epicondyle ( Fig. 1 ). This line coincided with the anterior border of the gracilis muscle. At the midpoint of this line, we drew a circle with a 3-cm radius divided into quadrants. Next, we made the incision following the circle contour. Then, we identified the perforating arteries by gentle dissection of the subcutaneous tissue, following them to the septum between the gracilis muscle and the vastus medialis/sartorius muscle. Most vessels identified in the intermuscular septum had muscular branches to the gracilis muscle and, in a few cases, to the sartorius muscle. These septal vessels gave off perforating branches to irrigate the skin and overlying subcutaneous tissue. The dissection continued deep into the femoral artery, which was identified as the arterial origin in all cases ( Fig. 2 ). The veins, usually two, were tributaries of the femoral vein, not of the great saphenous vein, which was close to the dissection site. The branch leading to the gracilis or sartorius muscle underwent ligation, preserving its proximal part. We did the same thing with the perforating veins leading to the skin, increasing the length of the vessels. We selected and documented vessels with an external diameter greater than 0.5 mm.

## Results

Table 1 shows the anthropometric data of the dissected cadavers, and Table 2 shows the flap vessel features.

**Table 1:** Anthropometric data

Thigh number	Age (years)	Side	Sex	Height (cm)	Weight (kg)	Thigh length (inguinal-medial femoral condyle) (cm)
**1**	56	Right	Male	176	70	38
**2**	56	Left	Male	176	70	38
**3**	62	Right	Male	174	66	36
**4**	62	Left	Male	174	66	36
**5**	56	Right	Male	165	88	36
**6**	52	Right	Male	172	78	35
**7**	52	Left	Male	172	78	35
**8**	56	Right	Male	176	84	35
**9**	52	Right	Male	150	43	35
**10**	52	Left	Male	150	43	35
**11**	64	Right	Male	167	61	30
**12**	64	Left	Male	167	61	30
**13**	58	Right	Male	170	70	30
**14**	58	Left	Male	170	70	30
**15**	59	Right	Female	169	95	30
**16**	59	Left	Female	169	95	30
**17**	42	Right	Male	182	76	35
**18**	42	Left	Male	182	76	35
**19**	66	Left	Male	175	74	26
**20**	58	Right	Male	175	86	34
**21**	58	Left	Male	175	86	34
**22**	92	Right	Female	164	68	28
**23**	92	Left	Female	164	68	28
**24**	47	Right	Female	160	65	29
**25**	47	Left	Female	160	65	29
**26**	36	Right	Female	165	70	33
**27**	36	Left	Female	165	70	33
**28**	92	Right	Female	163	55	32
**29**	92	Left	Female	163	55	32
**30**	85	Right	Female	157	42	29
**31**	85	Left	Female	157	42	29
**32**	38	Right	Female	160	62	29
**33**	38	Left	Female	160	62	29
**Mean**	**57.8**			**162.5**	**66.5**	**31.3**
**Maximum**	**92**			**182**	**95**	**38**
**Minimum**	**36**			**150**	**42**	**26**

**Table 2:** Vascular pedicle features

Thigh number	Pedicle length (mm)	Artery diameter (mm)	Vein diameter (mm)	Origin	Irrigated muscle	Quadrant	Variation
**1**	53	3.8	3	Femoral	Gracilis	Anterosuperior	
**2**	48	3.4	3.2	Femoral	Gracilis	Anterosuperior	
**3**	83	4.2	3.2	Femoral	Gracilis	Anterosuperior	
**4**	76	4.6	3.6	Femoral	Gracilis	Anterosuperior	
**5**	32	2.1	1.8	Femoral	None	Anteroinferior/Anterosuperior	No muscular branch
**6**	41	3.6	3.4	Femoral	Gracilis	Anteroinferior	
**7**	38	3.4	3.2	Femoral	Gracilis	Anteroinferior	
**8**	63	3.2	2.8	Femoral	Sartorius	Anterosuperior	Branch to the sartorius muscle
**9**	38	2	2	Femoral	Gracilis	Anterosuperior	
**10**	32	2.2	1.8	Femoral	Gracilis	Anterosuperior	
**11**	32	1.3	1	Femoral	Gracilis	Anterosuperior	
**12**	28	1.5	1.2	Femoral	Gracilis	Antroinferior	
**13**	78	1.6	1.4	Femoral	Gracilis	Anterosuperior	
**14**	45	2.5	2	Femoral	Gracilis	Anterosuperior	
**15**	84	1.8	1.8	Femoral	Gracilis	Anterosuperior	
**16**	76	1.6	1.6	Femoral	Gracilis	Anterosuperior	
**17**	38	1.3	1.2	Femoral	Gracilis	Anterosuperior	
**18**	40	1.4	1.2	Femoral	Gracilis	Anterosuperior	
**19**	32	1.6	1.4	Femoral	Gracilis	Anterosuperior	
**20**	40	2.2	2	Femoral	Sartorius	Anterosuperior	Branch to the sartorius muscle
**21**	38	2	2	Femoral	Sartorius	Anterosuperior	Branch to the sartorius muscle
**22**	62	1.5	1.5	Femoral	Gracilis	Anterosuperior	
**23**	60	1.3	1.5	Femoral	Gracilis	Anterosuperior	
**24**	58	2	1.8	Femoral	Gracilis	Antroinferior	
**25**	61	2.4	1.9	Femoral	Gracilis	Anteroinferior	
**26**	64	2.7	2.2	Femoral	Gracilis	Anterosuperior	
**27**	68	2.4	2.3	Femoral	Gracilis	Anterosuperior	
**28**	56	1.8	1.6	Femoral	Gracilis	Anterosuperior	
**29**	42	2.8	2	Femoral	Gracilis	Anterosuperior	
**30**	56	2.8	2.3	Femoral	Gracilis	Anterosuperior	
**31**	58	2.5	2.2	Femoral	Gracilis	Anterosuperior	
**32**	52	2	1.8	Femoral	Gracilis	Anterosuperior	
**33**	50	1.8	1.6	Femoral	Gracilis	Anterosuperior	
**Mean**	**50.6**	**2.3**	**2.0**				
**Maximum**	**84**	**4.6**	**3.6**				
**Minimum**	**28**	**1.3**	**1**				

**Fig. 3:**
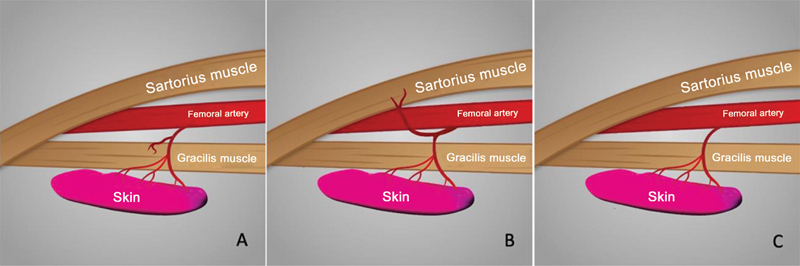
Arterial distribution patterns. Vessels with branches to the gracilis muscle ( **A** ). Vessels with branches to the sartorius muscle ( **B** ). Direct perforating vessels, with no muscular ramifications ( **C** ).

In all dissections (100%), we identified at least 1 artery accompanied by 2 veins and considered them viable for flap creation. All of these vessels were in the intermuscular septum between the gracilis and vastus medialis/sartorius muscles. In 29 (87.8%) of the 33 thighs, the main vessel was a septocutaneous artery with branches to the gracilis muscle; in 3 (9.1%) thighs, the septocutaneous arteries provided branches to the sartorius muscle; and 1 thigh (3%) had two direct septocutaneous arteries with no muscular branches ( Fig. 3 ).

**Fig. 4:**
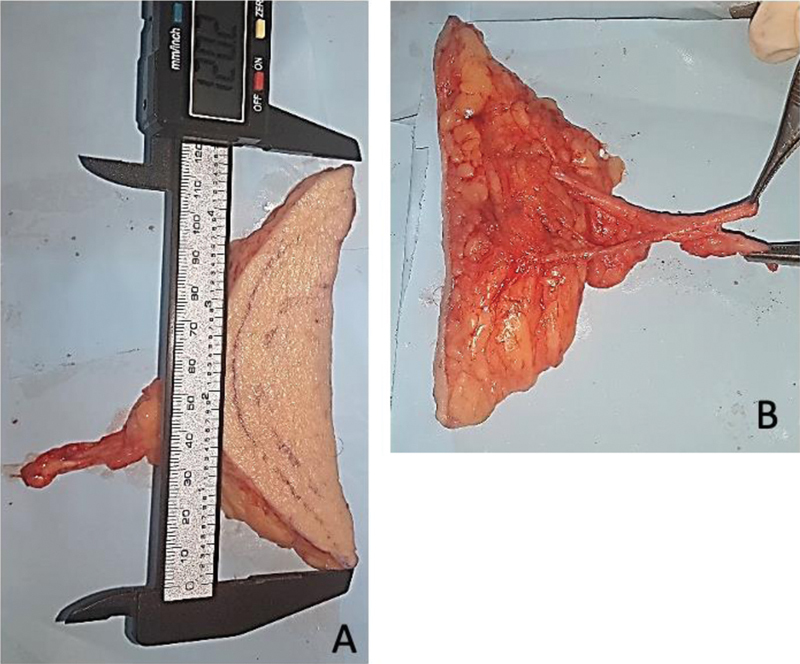
Isolated flap with an approximate length of 12 cm ( **A** ). Vascular pedicle ( **B** ).

All cases had at least 1 artery and 1 vein with an external diameter greater than 0.5 mm. The largest diameter was of 4.6 mm, and the smallest, of 1.3 mm. The mean artery diameter was of 2.3 mm. In general, there were 2 veins for each artery. The largest vein diameter was of 3.6 mm, and the smallest, of 1.0 mm, with a mean of 2.0 mm ( Fig. 4 ). The length of the vessels, from their origin in the femoral vessels to the smaller branches in the subcutaneous tissue, ranged from 28 mm to 84 mm, with a mean of 53.2 mm.

The statistical analysis started with the Kolmogorov-Smirnov parametric test to assess the normality of the distribution of the quantitative variables, and we concluded that there was a normal distribution. Regarding the distribution of the relative frequency (percentages) of qualitative factors, by applying the equality of two proportions test, we observed that the pedicle was in the anterosuperior quadrant in 84.8% of the dissected thighs. In addition, we noted that the gracilis was the muscle most commonly irrigated by this pedicle, corresponding to 87.9% ( *p* < 0.001) of the cases ( Tables 3 , 4 , respectively).

**Table 3:** Pedicle location at anteroinferior and anterosuperior quadrants

		n	%	*p* -value
**Quadrant**	Anteroinferior Anterosuperior	5 28	15.2% 84.8%	< 0.001

**Table 4:** Muscles irrigated by the vascular pedicle

		n	%	*p* -value
**Irrigated muscle**	Gracilis None Sartorius	29 1 3	87.9% 3.0% 9.1%	< 0.001 < 0.001

**Fig. 5:**
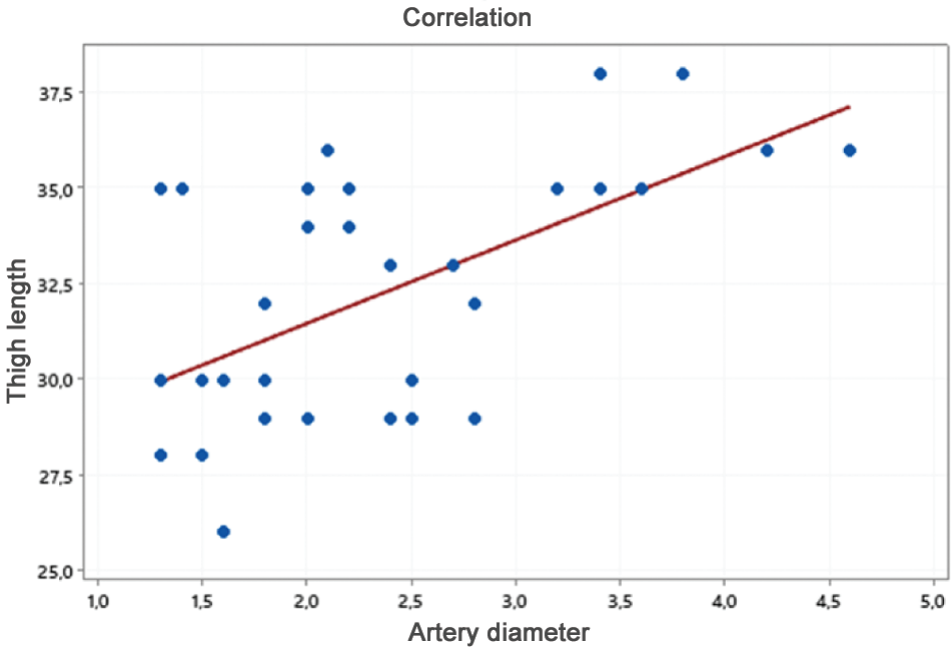
Relationship between thigh length and artery diameter.

The Pearson test analyzed the correlation among these variables. It revealed a significant and directly-proportional association between vessel diameter and thigh length ( *p* < 0.001) ( Fig. 5 ).

## Discussion

According to Giordano et al. 12 and Peek et al., 6 the gracilis muscle receives blood supply from a proximal main pedicle originating from the medial circumflex femoral artery and at least two distal secondary pedicles originating from the femoral or popliteal artery. Most of these primary and secondary pedicles have branches piercing the deep fascia and irrigating a considerable skin area over the gracilis muscle, corresponding to at least twice the width of this muscle. The anatomical characteristics of the main pedicle are well-known, resulting in reliable parameters to create gracilis or musculocutaneous flaps. However, the literature is scarce on secondary pedicles and their potential use as fasciocutaneous flaps.

The most common flaps are the proximal fasciocutaneous flaps of the thigh, irrigated by branches of the main pedicle of the gracilis muscle. Gracilis flaps are used widely in their pedicled and free forms. However, they compromise the main pedicle of the gracilis muscle and require care with the branches of the obturator nerve at the dissection site. The main vascular pedicle flap, harvested from the proximal medial region of the thigh, is in an area more susceptible to contamination and with limited access to postoperative dressings. 6

Scaglioni et al. 1 described some flaps of the anteromedial region in the distal region of the thigh based on the perforating musculocutaneous vessels crossing the vastus medialis. However, an adequate-length pedicle requires intramuscular dissection, and the scar area is more exposed compared with the main pedicle flap. Song et al. 10 and Algan and Tan 13 described a reliable flap originating from perforators of the deep femoral artery in this region, but often the patient's positioning makes it impossible to harvest it.

The proposed flap is on the medial side of the middle third of the thigh. After flap removal, primary closure of the donor area can be performed without the need for a skin graft. The scar is in a region of little exposure. The donor area is further away from the inguinal and perineal regions when compared with the main pedicle flap. Dressing change is more comfortable, and the prevention of contamination and infection is more reliable.

The mean arterial diameter in the current study was of 2.3 mm. According to Jacobson and Suarez, 14 this value is safe for vascular anastomoses in free flaps. The mean length of the pedicle was of 50.6 mm, which is adequate for free or pedicled flaps. However, in one of the dissections, we identified a pedicle shorter than 30 mm (28 mm), that is, a relatively short pedicle that would hinder anastomosis and depend on the area and the recipient's vessel.

Some interesting parameters found in the dissection included the following: all vascular pedicles were in the central region of the medial aspect of the thigh, within a circle with 6 cm in diameter, whose central point coincided with the anterior border of the gracilis muscle; the arterial origin was in the anterosuperior quadrant of the circle in 87.8% of the cases, and most of the vessels (87.8%) were septocutaneous vessels, with branches to the gracilis muscle. The greater saphenous vein requires care, as it is often included in the flap design and should be ligated and included in the flap if it is impossible to isolate.

**Fig. 6:**
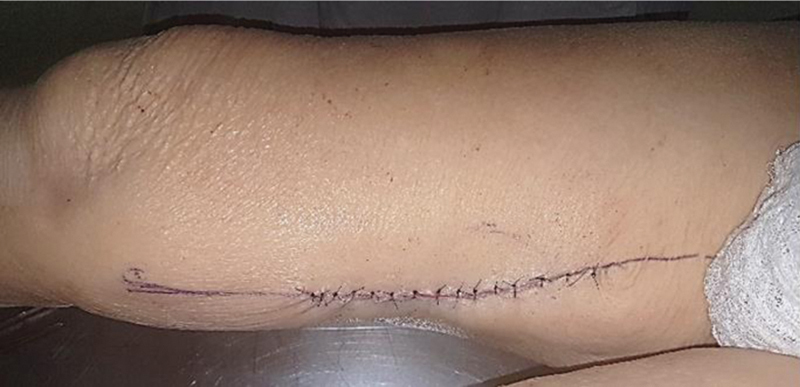
Primary closure of the donor area.

According to Giordano et al., 12 the width of the skin irrigated by the perforating vessels would correspond to at least twice the width of the underlying gracilis muscles. Closure of the donor area closure could be performed primarily, with relative ease, with no need for skin grafting ( Fig. 6 ).

The parameters found will enable the safe dissection and removal of a microsurgical flap. The skin of the medial region of the thigh is thinner and more versatile. A flap from this region is appropriate for covering small and medium-sized defects (shorter than 15 cm in diameter).

Case series 15 16 conducted in recent years have demonstrated the advantages of using perforating vessel flaps to obtain less bulky and malleable flaps; however, the flap herein presented was not described in any of these studies.

**Fig. 7:**
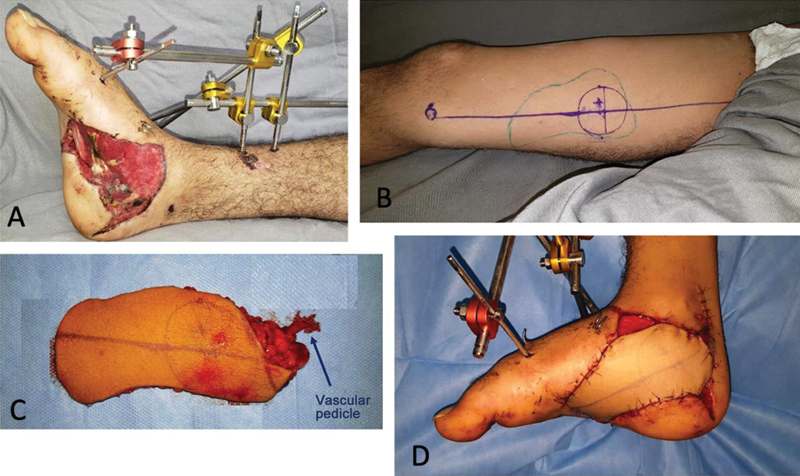
Operated case. Right foot with inferomedial defect coverage ( **A** ). Medial aspect of the right thigh with a drawing of the flap and location of the vascular pedicle ( **B** ). Isolated flap ( **C** ). Defect coverage with anastomosis in the posterior tibial vessels ( **D** ).

**Fig. 8:**
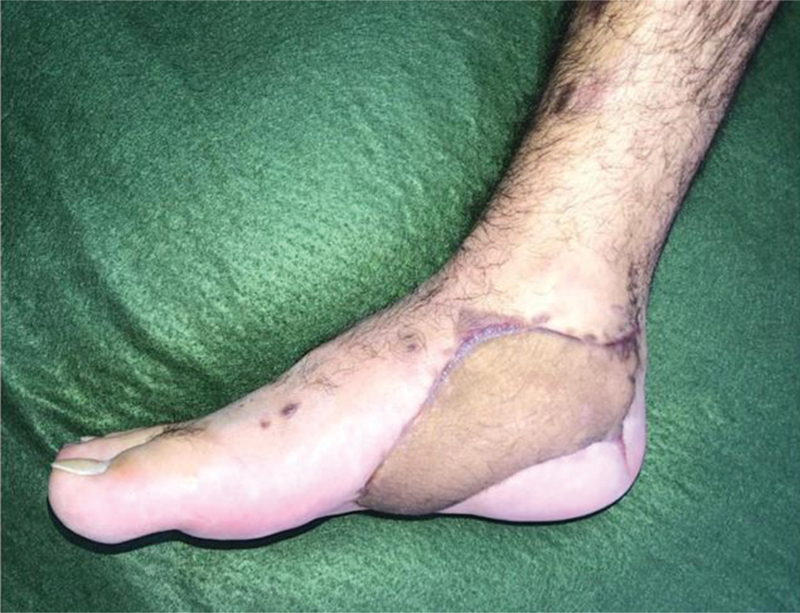
Operated case at three months of postoperative follow-up.

To demonstrate flap viability, we performed surgery on a young male patient who had been involved in a traffic accident and, in addition to multiple fractures on his right foot, had lost skin on the medial surface extending to the plantar surface, exposing tendons and the plantar aponeurosis ( Fig. 7 ). We performed microsurgical coverage with the flap described, with anastomosis of its vessels in branches and tributaries of the posterior tibial vessels. The procedure was successful, thus demonstrating technical viability ( Fig. 7 and Fig. 8 ).

In our opinion, one of the best indications for this flap would be upper limb skin coverage, which requires thin and malleable skin and donor vessels of smaller caliber. We intend to perform a series of cases using this flap type for later publication of the outcomes, showing the positive and negative points.

## Conclusion

We found the secondary pedicle of the gracilis muscle, which provides septocutaneous and perforating branches to the skin of the medial thigh, in all dissected thighs (100%). Therefore, this is a reliable option to obtain a fasciocutaneous flap from this region.

## References

[1] Scaglioni M F Fakin R M Giovanoli P Kuo Y R Kuo P J The lower medial thigh perforator (LMTP) flap for lower extremity reconstruction: Preliminary results Microsurgery 2016 36 06 474 479 10.1002/micr.30030 26806428

[2] Kim J T Kim S W Perforator Flap versus Conventional Flap J Korean Med Sci 2015 30 05 514 522 10.3346/jkms.2015.30.5.514 25931780 PMC4414633

[3] Fujino T Contribution of the axial and perforator vasculature to circulation in flaps Plast Reconstr Surg 1967 39 02 125 137 10.1097/00006534-196702000-00001 5335195

[4] Koshima I Soeda S Yamasaki M Kyou J The free or pedicled anteromedial thigh flap Ann Plast Surg 1988 21 05 480 485 10.1097/00000637-198811000-00015 2976584

[5] Wei F C Jain V Suominen S Chen H C Confusion among perforator flaps: what is a true perforator flap? Plast Reconstr Surg 2001 107 03 874 876 10.1097/00006534-200103000-00037 11304620

[6] Peek A Müller M Ackermann G Exner K Baumeister S The free gracilis perforator flap: anatomical study and clinical refinements of a new perforator flap Plast Reconstr Surg 2009 123 02 578 588 10.1097/PRS.0b013e3181956519 19182616

[7] Eom J S Sun S H Hong J P Use of the upper medial thigh perforator flap (gracilis perforator flap) for lower extremity reconstruction Plast Reconstr Surg 2011 127 02 731 737 10.1097/PRS.0b013e3181fed789 21285777

[8] Feng C H Yang J Y Chuang S S Huang C Y Hsiao Y C Lai C Y Free medial thigh perforator flap for reconstruction of the dynamic and static complex burn scar contracture Burns 2010 36 04 565 571 10.1016/j.burns.2009.07.005 19819077

[9] Zheng H Wang H Zhang F Yue S Anatomic basis of perforator flaps of medial vastus muscle Microsurgery 2008 28 01 61 64 10.1002/micr.20446 18085702

[10] Song Y G Chen G Z Song Y L The free thigh flap: a new free flap concept based on the septocutaneous artery Br J Plast Surg 1984 37 02 149 159 10.1016/0007-1226(84)90002-x 6713155

[11] Mathes S J Nahai F Classification of the vascular anatomy of muscles: experimental and clinical correlation Plast Reconstr Surg 1981 67 02 177 187 7465666

[12] Giordano P A Abbes M Pequignot J P Gracilis blood supply: anatomical and clinical re-evaluation Br J Plast Surg 1990 43 03 266 272 10.1016/0007-1226(90)90071-7 2350631

[13] Algan S Tan O Profunda femoris artery perforator flaps: a detailed anatomical study J Plast Surg Hand Surg 2020 54 06 377 381 10.1080/2000656X.2020.1801456 32762526

[14] Jacobson J H Suarez E L Microsurgery in anastomosis of small vessels Surg Forum 1960 11 243 245

[15] Abdelfattah U Power H A Song S Min K Suh H P Hong J P Algorithm for Free Perforator Flap Selection in Lower Extremity Reconstruction Based on 563 Cases Plast Reconstr Surg 2019 144 05 1202 1213 10.1097/PRS.0000000000006167 31397793

[16] Scaglioni M F Meroni M Knobe M Fritsche E Versatility of perforator flaps for lower extremity defect coverage: Technical highlights and single center experience with 87 consecutive cases Microsurgery 2022 42 06 548 556 10.1002/micr.30892 35475523

